# Standard Surgical Skin Markers Should Be Avoided for Intraoperative Vein Graft Marking during Cardiac and Peripheral Bypass Operations

**DOI:** 10.3389/fsurg.2016.00036

**Published:** 2016-06-20

**Authors:** Eric S. Wise, Joyce Cheung-Flynn, Colleen Marie Brophy

**Affiliations:** ^1^Department of Surgery, Vanderbilt University, Nashville, TN, USA; ^2^VA Tennessee Valley Healthcare System, Vanderbilt University, Nashville, TN, USA

**Keywords:** coronary artery bypass, vein graft disease, saphenous vein, surgical skin markers, brilliant blue FCF

## Introduction

One cause of early graft failure following coronary and peripheral vein grafting is kinking or mechanical twisting of the conduit. The blind tunneling necessitated in peripheral bypasses can lead to this consequence (1). In coronary artery bypass grafting (CABG), grafts to the posterior branches of the right or circumflex coronary arteries are placed to the back of the heart and are generally the longest aortocoronary grafts. These grafts are particularly prone to twisting and kinking. Additionally, single vein grafts to two or more coronary branches (“sequential vein grafts”) present special challenges with respect to maintenance of proper alignment (2, 3). The coronary vein graft failure rate at 1 year in the PRoject of *Ex vivo* Vein graft ENgineering via Transfection IV (PREVENT IV) cohort was 25%, suggesting that early graft failure is a significant problem (4, 5). The incidence of vein graft failure due to twisting or kinking is not well studied, though some reports estimate that obstructing lesions, including but not limited to twisting or kinking, account for 15–25% of early (<1 year) graft failures (6–8).

Preparation and preservation techniques have advanced the field of solid organ allotransplantation, but vein graft failure rates suggest room for improvement in the handling of saphenous vein (SV) harvested for autotransplantation. Interventions for *ex vivo* graft treatment, including appropriate choice of preservation solution and minimization of manual pressure distension *via* chemical vasodilators such as papaverine, have been proposed (9–12). Additionally, the “no-touch” technique has emerged as a method of minimizing detrimental handling of graft during harvest with adventitial preservation (4, 13–15).

Marking of the SV graft using a surgical skin marker in an “off-label” fashion represents another such preparation technique and is the most commonly employed method of preventing graft torsion (16). This marking allows for the maintenance of continuous alignment along the length of the vein in the same longitudinal plane. These lines provide a visible guide for surgeons to use during conduit routing and tunneling to avoid mechanical obstructions related to vein alignment (17). While operating rooms have adapted less traumatic methods of vein harvest, preservation, and distension, surgical skin marker use remains ubiquitous as the primary method to maintain graft orientation. The use of surgical skin markers during graft preparation is toxic to the tissue and impairs physiologic function (16). We argue that the data support avoidance of standard surgical skin markers for marking on vascular tissue; We further contend that brilliant blue FCF (for coloring food) may represent a viable alternative dye for intraoperative graft marking.

## Current Status of Graft Marking

Also known as crystal violet, gentian violet has emerged as the most common dye used in surgical skin marking in the United States, replacing methylene blue in the mid-1990s (18). Most commercially available markers approved for use to mark skin contain 10% gentian violet dye in an isopropyl alcohol solvent, at up to 50% by weight (16). In addition to off-label use on vein grafts, gentian violet-based surgical skin markers are routinely used to mark other human tissue intraoperatively, including tendon and eye tissue such as donor cornea and anterior lens capsule (19–22). Influence of the gentian violet marker on residual human hamstring tendon following graft placement during anterior cruciate ligament reconstruction was assessed using an *in vitro* live–dead assay. The study found that marking caused pronounced cell death and inhibited tenocyte migration, representing a total failure of explant vigor (20). While the effect on clinical outcome has not yet been realized, the authors recommended minimization of tendon marking. In ocular tissue, gentian violet pen markings are often made on a microscale on transplanted corneal tissue to maintain orientation and to anterior lens capsular tissue to facilitate intraoperative visualization in cataract surgery (19, 22). To this effect, there is evidence that suggests the dye may be toxic to donor corneal endothelium in Descemet-stripping automated endothelial keratoplasty and to anterior lens epithelial cells in corneal surgery.

Few studies have addressed the mechanism by which gentian violet affects mammalian cells, at the molecular level. On the one hand, gentian violet blocks the activity of nicotinamide adenine dinucleotide phosphate oxidases, avoiding the generation of superoxide radicals and subsequent inflammation (23). However, gentian violet also has been shown to bind to intracellular deoxyribonucleic acid, precluding DNA replication and excision repair (24).

The effect of gentian violet on human vessels is controversial. It was previously reported that when applied to human venous tissue, gentian violet acutely preserved physiologic functional responses better than methylene blue, as measured on an organ bath (16, 18). More specifically, these studies showed that topical gentian violet application did not significantly impair smooth muscle contractile strength induced by potassium chloride and phenylephrine stimuli or vasodilatory response to cholinergics and calcium channel blockers; dilatory responses to sodium nitroprusside, however, were significantly decreased (18). During the course of physiologic studies using human SV surgical remnants from CABG patients, our laboratory observed that tissue segments with signs of surgical skin marker containing both gentian violet and isopropyl alcohol displayed markedly impaired responses in the muscle bath. Human SV samples were marked with the standard surgical skin markers and, after equilibration on an organ bath, were challenged with vasoactive agents. Compared with unmarked control segments, the marked segments demonstrated impaired contractility in response to potassium chloride and phenylephrine agonists and decreased endothelial-dependent relaxation in response to carbachol, a cholinomimetic (16). The cytotoxic effects of topical gentian violet dye on cultured human umbilical venous smooth muscle cells has recently been reported. Brief exposure of these cells to topical gentian violet dye at subclinical concentrations leads to cytologic fixation. Additionally, this treatment leads to enhanced dead cell protease release relative to control cells, as determined *via* the CytoTox-Glo assay (25).

Isopropyl alcohol is a clear, colorless liquid with many commercial applications, including cosmetics, antifreezes, and cleaners. It is the solvent used for gentian violet in current surgical skin markers. Topical application of isopropyl alcohol on a vessel carries the risk of systemic absorption, although subsequent hepatic conversion to acetone *via* alcohol dehydrogenase may reduce the harm. Unlike other alcohols, isopropyl alcohol itself, rather than its product of oxidation, is responsible for the toxic effects (26, 27).

The physiologic effect of isopropyl alcohol on human SV was also investigated in our laboratory and reported in 2011 (16). As seen with standard surgical skin marking, contractile responses of human SV rings in response to potassium chloride and phenylephrine challenge were decreased in tissues briefly preserved in 50% aqueous isopropyl alcohol, when assessed on an organ bath (16). Endothelial-dependent relaxation was also impaired with isopropyl alcohol-preserved tissues. Diminished contractile responses to potassium chloride have been associated with decreased viability of human SV. Evidence of isopropyl alcohol-induced cellular damage has been further supported by staining with trypan blue, a vital stain used to detect dead tissue. Cultured human umbilical venous smooth muscle cells, after a 15-min exposure to 50% isopropyl alcohol, histologically demonstrated significantly increased uptake of trypan blue. Additionally, isopropyl alcohol exposure, as seen with gentian violet, led to an increase in cytotoxicity as determined *via* the CytoTox-Glo assay (25). Most concerning, this pattern of acute cellular injury to the conduit may portend a poor graft performance, leading to thrombosis and acceleration of neointimal hyperplasia (28–30).

## Proposed Alternative

**Figure 1:**
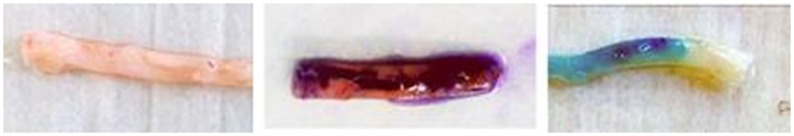
**Ink markings on human saphenous vein graft**. *Left* – unmarked human saphenous vein; *Middle* – human saphenous vein marked with a standard surgical skin marker (gentian violet dye and isopropyl alcohol solvent); *Right* – human saphenous vein marked with brilliant blue FCF-based marking pen (FCF dye and glycerol solvent).

A common food colorant, FCF is a triarylmethane water-soluble dye that is found to be safe in commonly ingested doses (31). Topically, FCF appears as a distinct bright blue similar to methylene blue and, like current vein graft dyes, is easily distinguishable in the intraoperative setting from blood, irrigants, and surrounding muscle and connective tissue (Figure 1). As a structural analog of brilliant blue G, it was plausible that FCF too may inhibit P2X_7_ receptors, cell surface receptors whose activation is induced by the trauma of intraoperative graft manipulation. Our laboratory has shown that these receptors are implicated in proliferation, migration, and apoptosis of vascular smooth muscle cells (32). Due to its established safety profile and potential for amelioration of preparation-induced conduit injury *via* P2X_7_ receptor inhibition, FCF represented a practical candidate for a substitute vein graft dye (25, 32).

Evidence of FCF-governed P2X_7_ inhibition has been reported, primarily from our laboratory. The P2X_7_ agonist, 2′(3′)-*O*-(4-benzoylbenzoyl)adenosine-5′-triphosphate (BzATP), was added to an organ bath containing suspended unprepared human SV rings left as untreated control or pretreated with a topical FCF. The FCF-treated rings demonstrated reduced BzATP-induced, P2X_7_-governed contractile responses, suggesting that P2X_7_ receptors were inhibited (32). P2X_7_ receptor activation leads to increases in intracellular calcium concentrations (33). Further evidence supporting the proposed mechanism of action of FCF was recently reported, as FCF pretreatment of rat aorta prevented intracellular calcium flux governed by BzATP-induced P2X_7_ activation, as detected by a FluoroPlex apparatus (25).

We have also examined the topical effect of FCF on vascular conduit. Human SV samples were obtained from CABG patients prior to preparation or preservation, and baseline physiologic responses were determined in an organ bath (32). The decrement in endothelial-dependent relaxation observed after 2 h of preservation in heparinized Plasma-Lyte A was mitigated by the addition of aqueous FCF to the preservation solution (32). Moreover, in some human SV samples that were initially unresponsive to a depolarizing KCl stimulus, FCF restored smooth muscle contractility and hence, functional viability (32). Exposure of cultured human umbilical venous smooth muscle cells to clinically applicable doses of topical FCF has recently been shown to lead to neither increased trypan blue staining relative to control nor increased dead cell protease release *via* the CytoTox-Glo assay, in contrast to the effects observed with topical gentian violet and isopropyl alcohol (25).

In addition to its effects on acute physiologic responses and cellular viability of vascular tissue, we have also reported the influence of FCF treatment on intimal hyperplasia (32). Unmanipulated human SV rings were treated for 2 h in solution with and without FCF, and maintained in organ culture over 14 days. While a significant increase in intimal thickness was noted among untreated veins, this was mitigated in the FCF-treated vein group (32). FCF, as a topical vein graft treatment in a rabbit external jugular–carotid artery interposition model, led to reduced neointimal growth relative to control grafts (34). Finally, platelet-derived growth factor-induced migration and proliferation were examined in A7r5 smooth muscle cells. FCF pretreatment abrogated both cellular migration and proliferation (34).

## Conclusion

The “off-label” use of surgical skin markers have been shown to impair physiologic responses in human SV graft and to be cytotoxic. Surgeons should consider minimizing or eliminating the off-label use of surgical skin markers for vein marking. FCF is a plausible alternative dye for graft marking in that it preserves endothelial and smooth muscle function and inhibits neointimal growth in human SV. Moreover, FCF is also the only dye that is currently approved by the FDA for use in marking human SV.

## Author Contributions

EW contributed to research and writing of the manuscript. JC-F contributed to research and critical revision of the manuscript. CB oversaw all respects of the research and composition of the manuscript. All authors gave final approval.

## Conflict of Interest Statement

JC-F and CB have a proprietary interest in VasoPrep Surgical, Inc. EW has no conflicts of interest to disclose.
